# The Effect of Music Intervention on Attention in Children: Experimental Evidence

**DOI:** 10.3389/fnins.2020.00757

**Published:** 2020-07-24

**Authors:** Yuka Kasuya-Ueba, Shuo Zhao, Motomi Toichi

**Affiliations:** ^1^Department of Human Health Science, Graduate School of Medicine, Kyoto University, Kyoto, Japan; ^2^Institute of Psychology, Shenzhen Key Laboratory of Affective and Social Neuroscience, Shenzhen University, Shenzhen, China; ^3^The Organization for Promoting Neurodevelopmental Disorder Research, Kyoto, Japan

**Keywords:** music intervention, attention, children, cognitive function, cognitive development

## Abstract

Although music has been utilized as a therapeutic tool for children with cognitive impairments, how it improves children’s cognitive function remains poorly understood. As a first step toward understanding music’s effectiveness and as a means of assessing cognitive function improvement, we focused on attention, which plays an important role in cognitive development, and examined the effect of a music intervention on children’s attention. Thirty-five children, aged 6 to 9 years, participated in this study, with data from 29 of the children being included in the analysis. A single 30-minute interactive music intervention was compared with a single 30-minute interactive video game intervention accompanied by computer-generated background music using a within-subjects repeated-measures design. Each intervention was implemented individually. Participants completed a standardized attention assessment, the Test of Everyday Attention for Children, before and after both interventions to assess changes in their attentional skills. The results indicated significant improvement in attention control/switching following the music intervention after controlling for the children’s intellectual abilities, while no such changes were observed following the video game intervention. This study provides the first evidence that music interventions may be more effective than video game interventions to improve attention control in children, and furthers our understanding of the importance of music interventions for children with attention control problems.

## Introduction

Music is a powerful sensory stimulus that produces physiological, psychological, and social effects (Hodges, 1996; Davis et al., 2008; Thaut and Hoemberg, 2014; Wheeler, 2015). The clinical application of music, when utilized in a purposeful and systematic way, reportedly enhances development in special needs children (Robb, 2003a). Although the effectiveness of music interventions on communication, social skills, and emotional development in children has been well documented, and therapeutic effects of music on cognition in adults have also been shown – for example, on episodic memory in patients with dementia (Irish et al., 2006) and traumatic brain injuries (Särkämö et al., 2008) focused attention in stroke patients (Särkämö et al., 2008) and sustained attention in adults with cognitive deficits (Gregory, 2002) evidence pertaining to music’s effects on attention and other cognitive functions in children is limited, and the mechanisms by which music improves cognitive functions in children remain poorly understood.

Several studies have reported that music interventions may have a positive impact on attention. It has been proposed that music itself contains therapeutic factors that enhance attention skills; for example, rhythmic patterns drive attention focus, and musical elements such as rhythm, melody, and harmony provide multidimensional stimuli that facilitate switching attention (Gardiner, 2005; Thaut and Gardiner, 2014). The perception of rhythmic, melodic, harmonic, and dynamic patterns in music may influence the focus and organization of the flow of our attention (Thaut et al., 2008). Attention is a fundamental skill for good cognitive functioning and thus plays an important role in cognitive, social, and communication development (Muris, 2006; van de Weijer-Bergsma et al., 2008; Cornish and Wilding, 2010; Matson et al., 2010; Rueda et al., 2010; Janzen and Thaut, 2018). Several studies examining preterm infants have found that individual differences in attentional problems early in development can predict later cognitive and behavioral functioning (van de Weijer-Bergsma et al., 2008). Furthermore, attention control in school-age children is positively correlated with academic achievement (Muris, 2006; Rueda et al., 2010). Attention skills develop in a stepwise fashion from infancy through engagement with one’s environment (Ruff and Rothbart, 2001; Atkinson and Braddick, 2012) exploring the external world, and orienting to, shifting between, and maintaining focus on events, objects, and tasks (van de Weijer-Bergsma et al., 2008). When the development of these basic skills is hindered, there may be adverse effects on cognitive, social, and communication skills; thus, it is important to learn more about the potential therapeutic effects of music intervention on children’s attention.

Only a few mixed studies have reported on the effects of music intervention on children’s attention. For instance, Wolfe and Noguchi (2009) examined the effects of music on sustained attention in 5-year-old children using vigilance tasks that required verbal and motor responses. The children listened to a musical or spoken story with or without distraction, and results indicated that the children listening to a musical story in the distraction condition performed significantly better than the children listening to a spoken story with distraction. Morton et al. (1990) investigated 10-to-12-year-old children using a verbal dichotomous listening task that was preceded by exposure to music and exposure to silence. The authors observed reduced distractibility in the children in the directed-report task and increased memory capacity in the children in the free-report task, after being exposed to music.

Other studies have investigated music therapy in children with special needs, where music is used as a therapeutic tool. For example, Lee (2006) reported a case study of a 5-year-old child with autism spectrum disorder (ASD) who showed better attention span in behavioral observations after 20 sessions. Kasuya (2011) reported a case study of an 8-year-old boy with attention deficit/hyperactive disorder (ADHD) whose attentive behaviors improved during music therapy sessions (measured with behavioral observations), and sustained attention and impulsive behaviors also improved on a continuous performance task after 24 sessions. Robb (2003b) investigated attentive behavior among six preschool children with visual impairments and found that the children’s attentive behaviors were significantly more frequent during music-based sessions than during play-based sessions. Knox et al. (2003) reported that a brain injured adolescent showed improvements in alternating attention following intervention from a Musical Attention Training Program, which required participants to switch concentration between a melodic line and a drum track. Pasiali et al. (2014) examined the effectiveness of a standardized music therapy technique, Musical Attention Control Training (MACT; Thaut and Gardiner, 2014) in nine 13-to-20-year-old adolescents with varying severity of neurodevelopmental delays. The authors found positive improvements in selective attention and attention control on an attention test battery after eight group sessions. Abrahams and van Dooren (2018) also found positive trends in attention tests for children with attention deficits after six weekly MACT sessions. They pointed out, however, that the sample size was small, with only two participants in each of the experimental and control groups, and that attention outcomes varied with individual participants. Although these studies have yielded limited and mixed results regarding the effects of music interventions on children’s attention, they suggest that music may have a positive impact. However, none of the previous studies evaluated the influence of music interventions on different types of attention (i.e. sustained attention, selective attention, and attention control) in multiple participants.

More recent longitudinal studies conducted in children have focused on the impact of music training on non-musical cognitive functions (Miendlarzewska and Trost, 2014; Benz et al., 2016; Dumont et al., 2017; Sala and Gobet, 2017). For example, Schellenberg (2004) found greater increases in full-scale IQ after one year of keyboard or voice lessons. Barbaroux et al. (2019) evaluated the impact of an 18-month classic music training program on the cognitive functions of children from low socio-economic backgrounds and found significant improvements in general intelligence, processing speed, concentration abilities, and reading precision. On the other hand, while Linnavalli et al. (2018) found that music playschool over the course of two school years significantly improved phoneme processing and vocabulary skills, they did not see improvements in non-verbal reasoning or inhibitory control. Yang et al. (2014) found that long-term music training significantly improved musical achievement and second language development, but there was no improvement in first language or mathematics. Nan et al. (2018) found that 6 months of piano training significantly improved auditory word discrimination compared to a reading training or a control group, however, no differences were found in general cognitive measures, including attention, which improved equally among the three groups. Although these studies have reported mixed evidence, their findings suggest that long-term music engagement intended to improve musical skills has beneficial effects on non-musical functions, including intellectual abilities.

Currently it is unknown whether music interventions designed to improve children’s attention are effective. Thus, in this study we examined the effect of a short-term music intervention (i.e., a single 30-minute trial) on attention in children using a therapeutic technique, Musical Attention Control Training (Thaut and Gardiner, 2014) as a first step toward broadly investigating the effectiveness of music therapy for improving cognitive functions. To investigate the pure effect of the music intervention, we used an active control intervention with similar features as the music intervention, except with no live music. The specific aims were: (1) to investigate the effects of a music intervention on children’s attention and (2) to assess whether specific subtypes of attention (i.e., sustained attention, selective attention, attention control/switching, and divided attention) are responsive to this music intervention. Although certain factors, such as intelligence and ADHD traits, may affect attentional performance (Manly et al., 2001; Imada et al., 2003; Cornish and Wilding, 2010; Hurford et al., 2017; Mous et al., 2017) no previous investigations have controlled for these influences. Thus, we investigated changes in children’s attention skills (behavior measured before and after a music intervention as an experimental task and a video game intervention as a control task) using a standardized attention test, alongside the ADHD Rating Scale (ADHD-RS; Ichikawa and Tanaka, 2008) and Raven’s Colored Progressive Matrices (Raven, 1998) which were administered at the start of the experiment. Our goal was to offer initial information regarding the effects of a music intervention on children’s cognitive functions and to provide evidence on the feasibility of music interventions for future clinical research with children who have cognitive impairment.

## Materials and Methods

### Participants

Participants were recruited through advertisements for healthy children, aged 6 to 9 years, without a history of serious neurological illness (e.g., brain injuries), as confirmed via a parental report (*n* = 35). The above-mentioned age range was chosen because the Test of Everyday Attention for Children (Manly et al., 1999) used in this study has been standardized and normed for children between the ages of 6 and 16. In addition, to ensure that the developmental stage of the participants’ working memory and self-awareness were as similar as possible, children under 10 years of age were targeted. The sample size was determined with reference to previous studies (Schlaug et al., 2005; Tamm et al., 2010; Scott, 1992) which examined the positive impact of interventions on children’s cognitive functioning. Parents contacted the first author by phone or e-mail to schedule 2 days for participation in this study. On the first day of the experiment, with their parents present, participants were interviewed by an expert child psychiatrist to identify any signs of developmental disorders. All 35 participants completed the experimental procedure, however, six participants were excluded from statistical analysis: three were excluded for possibly having developmental disabilities, as assessed by the child psychiatrist and their scores on the ADHD-RS as completed by their parents; the other three were excluded because their TEA-Ch scores were extreme outliers (more than two standard deviations). Therefore, data from 29 participants were analyzed, including five pairs of siblings. Table 1 shows the demographic characteristics of the participants who were included in our statistical analyses.

**TABLE 1:** Demographic Characteristics of the Participants (*n* = 29).

**Male:Female**	**15:14**	
	**Mean (SD)**	**Range**
Age	7.4 (1.3)	6.0 to 9.11
ADHD-RS	8 (7.7)	0 to 36
RCPM	28.3 (4.9)	19 to 35

### Procedure

An experimental within-subjects repeated-measures design was used. The experimenter administered the TEA-Ch to the children prior to and after participating in an experimental task (i.e., music intervention) and a control task (i.e., video game intervention), which were conducted individually on separate days at least one week apart in a quiet office at the university.

On the first day of each experimental procedure, the participant and the participant’s parent entered the office, and the experimenter verbally explained the study and provided individual written informed consent forms; all participants assented to participate and all parents consented to having their child(ren) participate by signing the consent document. Next, the parent completed the ADHD-RS while the participant was administered the RCPM by the experimenter. Then, the participant and the parent were interviewed by the child psychiatrist. After the interview, the parent left the office and the participant was administered the TEA-Ch by the experimenter and an assistant. The TEA-Ch has two parallel versions, version A (pre-test) and version B (post-test), that allow for assessing test-taker improvement; thus, version A was administered prior to a 30-minute music intervention or video game intervention and version B was administered after the intervention and a brief break (about 10 min). If the participant completed the music intervention on the first day, they completed the video game intervention on the second day, or vice versa. Intervention order was counterbalanced by alternate assignment, where half of the participants completed the music intervention first, and the other half completed the video game intervention first. On the second day, the participant was administered the TEA-Ch version A, prior to the 30-minute music or video game intervention, and version B after the intervention and a brief break. Total time required for the experiments was approximately two-and-a-half to 3 h on the first day and two to two-and-a-half hours on the second day. During test and intervention implementation, participants were videotaped with their parents’ permission.

### Measures

#### ADHD Rating Scale-IV (ADHD-RS)

The ADHD-RS, originally created by DuPaul et al. (1998) and translated into Japanese by Ichikawa and Tanaka (2008) is an 18-item scale that takes approximately 5 min to complete. It measures ADHD symptoms according to the DSM-IV diagnostic criteria (American Psychiatric Association, 1994). Each of the 18 items is scored from 0 to 3: 0 = none (never or rarely); 1 = mild (sometimes); 2 = moderate (often); 3 = severe (very often). We used the home version, where a parent reports the frequency of symptoms over the past 6 months, and obtained the total score by summing all scores. The maximum score possible is 54 and the minimum is 0, with higher scores indicating greater severity of ADHD. We referred to this score when interviewing the participants and their parents and used it as a covariate to control for participants’ ADHD traits.

#### Raven’s Colored Progressive Matrices (RCPM)

The RCPM (Raven, 1998) is a fast and easy-to-administer test of non-verbal reasoning used to verify that participants had no intellectual disabilities. It contains 36 items in three sets to assess general intellectual development for children aged 5 to 11 years and adults. Each item presents participants with an incomplete design and six alternatives; they must choose the one that best completes the design. We obtained a total score by summing the items that were correctly answered. The maximum score is 36 and the minimum is 0, with higher scores indicating better performance. We used this score as a covariate to control for participants’ intellectual abilities.

#### Test of Everyday Attention for Children (TEA-Ch)

The TEA-Ch was created by Manly et al. (1999) and includes nine subtests of different types of skills (e.g., sustained attention, selective attention, attention control, and ability to inhibit verbal and motor responses). This assessment, which is conducted on a one-to-one basis, has been standardized and normed for children and adolescents between the ages of 6 and 16. The assessment takes about one hour to complete; thus, we used only the first four subtests to briefly measure each attentional factor and dual task performance (Manly et al., 1999) within the allotted time to avoid fatiguing the children. The four subtests were completed in 20 to 30 min and yielded seven raw scores, which were then converted to age-scaled scores by using the appropriate normative table provided in the test manual (Manly et al., 1999). The age-scaled scores for each subtest range from 1 to 20, with 20 representing the best performance. The age-scaled scores had a mean of 10 and standard deviation of 3. The tool has two parallel versions (version A and B) to allow for test-retest. Test-retest reliability coefficients for the subtests ranged from 0.57 to 0.87.

The four subtests were (1) “Sky Search” for selective/focused attention, (2) “Score!” for sustained attention, (3) “Creature Counting” for attention control/switching, and (4) “Sky Search Dual Task” (“Sky Search DT”) for sustained-divided attention.

(1) *Sky Search.* In this task, the participant is given a large sheet filled with spaceship pairs and distractor dissimilar spaceships and is asked to find and circle pairs of spaceships that are the same as quickly as possible. The second part of the task, which has no distractor items, is used as a control for motor speed differences. Scoring involves counting the number of correct pairs circled and the time taken; a test administrator records time per target by dividing the latter by the former, after which a motor control score is subtracted from the time per target score. Three scores (“accuracy,” “time per target,” and “attention score”) are obtained to measure the child’s selective/focused attention skill.
(2) *Score!* This task involves silently counting the number of shooting sounds a participant hears (ranging from 9 to 15) on a 6-minute audio track, without using fingers and with long gaps between sounds. The raw score is obtained by giving one point for each of the 10 trials counted correctly. The “accuracy” score is obtained to measure the child’s sustained attention skill.
(3) *Creature Counting.* The participant counts the number of creatures in a burrow in the cue book, following a visual pathway—counting up when the arrow points up and down when it points down. Time taken and accuracy for each of the seven trials are recorded and two scores (“accuracy” and “speed”) are obtained to measure the child’s attention control/switching skill.
(4) *Sky Search DT.* The participant performs the subtests “Score!” and “Sky Search” at the same time, meaning they visually find spaceship pairs as quickly as possible while auditorily keeping count of shooting sounds on the audio track. Time taken is recorded, correct responses for both tasks are counted, and a dual score is calculated. The “decrement” score is obtained to measure the child’s sustained-divided attention skill.

### Tasks

The video game intervention was adopted as a control task administered under similar conditions as the experimental task except with no live music. Both tasks (1) involved upper extremity movement, (2) were interactive with the experimenter, and (3) were easy to play and child friendly. Furthermore, most children find both video games and musical instruments enjoyable, and both activities tend to grab children’s attention. For the video game intervention, participants played a bowling video game from Nintendo Wii Sports with the experimenter, which was a straight-up, 10-pin, 10-frame game with standard rules for two players. The players took turns holding and swinging the Wii remote in one hand to roll the ball with sufficient force and aim to get a good score. Three or four sets of the game were played in 30 min.

Musical Attention Control Training (MACT; Thaut and Gardiner, 2014) was used as the experimental music intervention. The MACT involves ‘structured active or receptive musical exercises involving precomposed performance or improvisation in which musical elements cue different musical responses to practice attention functions’ (Thaut and Gardiner, 2014 p. 257). In this study, the participant played percussion instruments with the experimenter who sang, played a keyboard, or played percussion. During the first 10 min, the experimenter held a hand drum in each hand, facing the participant, who held a mallet in each hand, and asked the participant to hit the drums held up alternately by the experimenter with the left and right hand, as the experimenter sang a simple, original “Let’s play the drum” song. In the next 10 min, the participant played three kinds of percussion instruments (congas, cymbal, and Remo Tubano) as follows: (1) played them freely while the experimenter played the keyboard; (2) played an appropriate one as the experimenter played high, middle, or low range on the keyboard, following instructions (e.g., asked to play the cymbal when hearing high notes); and (3) played by matching how the experimenter played on the keyboard (e.g., played loudly when the experimenter played loudly and stopped playing when the experimenter stopped). In the last 10 min, the experimenter and participant faced each other and imitated each other’s rhythmic patterns while taking turns on the same percussion instruments set between them. These activities implemented in the music intervention were designed according to our participants’ ages to easily produce rhythmic responses against a clear, steady beat (Thaut and Gardiner, 2014).

### Data Analysis

After excluding 6 of 35 participants (as described previously), data from the remaining 29 participants were analyzed using IBM SPSS Statistics, version 24.0. Mean TEA-Ch scores under each condition were calculated for each participant. First, seven raw scores were converted to age-scaled scores by using the appropriate normative table from the test manual (Manly et al., 1999). Thus, raw data were transformed to a normal distribution, effectively removing the influence of age (Manly et al., 2001) this was done to examine whether attentional performance, including selective/focused attention, sustained attention, attention control/switching, and sustained-divided attention were influenced by the music or video game interventions. Since the four attentional performance subtests do not equally contribute to the scoring, we investigated whether attentional performance was modulated by the music or video game interventions under selective/focused attention, sustained attention, attention control/switching, and sustained-divided attention, separately. Thus:

(1) In “Sky Search,” we examined “accuracy” (subscore 1; s1) “time per target” (subscore 2; s2) and “attention score” (subscore 3; s3) during selective/focused attention to assess whether attentional performance was modulated by the music or video game interventions. Score differences were analyzed using a repeated-measures analysis of variance (ANOVA) with Task (music, game), Time (pre, post), and TEA-Ch Score (s1, s2, s3) as within-participant factors.
(2) In “Score!,” we examined “accuracy” (subscore 4; s4) during sustained attention to assess whether attentional performance was modulated by the music or video game interventions. Score differences were analyzed using a repeated-measures ANOVA with Task (music, game), Time (pre, post), and TEA-Ch Score (s4) as within-participant factors.
(3) In “Creature Counting,” we examined “accuracy” (subscore 5; s5) and “speed” (subscore 6; s6) during attention control/switching to assess whether attentional performance was modulated by the music or video game interventions. Score differences were analyzed using a repeated-measures ANOVA with Task (music, game), Time (pre, post), and TEA-Ch Score (s5, s6) as within-participant factors.
(4) In “Sky Search DT,” we examined “decrement” (subscore 7; s7) during sustained-divided attention to assess whether attentional performance was modulated by the music or video game interventions. Score differences were analyzed using a repeated-measures ANOVA with Task (music, game), Time (pre, post), and TEA-Ch Score (s7) as within-participant factors.

If an interaction was significant, a follow-up simple main effect analysis (i.e., assessing the effect of each independent variable at each level of the other independent variable) was conducted to interpret the result (*p* < 0.05, uncorrected for multiple tests).

Finally, to avoid the influence of individual characteristics, including participants’ intellectual abilities and ADHD traits during selective/focused attention, sustained attention, attention control/switching, and sustained-divided attention, score differences were analyzed using repeated-measures analysis of covariance (ANCOVA) with Task (music, game), Time (pre, post), and TEA-Ch Score (e.g., s1, s2, s3) as within-participant factors, and participant RCPM scores, ADHD-RS scores, and RCPM scores × ADHD-RS scores as covariates.

Additionally, we examined whether attentional performance differences were influenced by task order. The results indicated that attentional performance was not modulated by task order during selective/focused attention, sustained attention, attention control/switching, or sustained-divided attention (see Supplementary Material).

## Results

Table 2 shows the averaged pre and post age-scaled scores for each of the seven subscores (s1–s7) from the four TEA-Ch subtests. Table 3 shows the difference in Time condition between pre and post during music and video game intervention.

**TABLE 2:** Mean Scores for Each Subtest of TEA-Ch at Pre/Post Using Age-Scaled Scores.

**TEA-Ch subscores**		**Music intervention (*n* = 29)**		**Video game intervention (*n* = 29)**	
		
		**Mean (SD)**			
		
		**Pre**	**Post**	**Pre**	**Post**
**Selective/focused attention**					
s1	Sky Search Accuracy	11.4 (2.4)	12.1 (2.6)	11.3 (2.5)	11.4 (3.1)
s2	Sky Search Time per Target	12.0 (3.1)	13.3 (2.6)	12.1 (3.1)	13.6 (2.6)
s3	Sky Search Attention Score	11.3 (3.1)	12.6 (2.7)	11.6 (3.5)	12.7 (3.1)
**Sustained attention**					
s4	Score! Accuracy	10.6 (3.8)	10.6 (3.0)	10.1 (3.2)	9.4 (3.9)
**Attention control/switching**					
s5	Creature Counting Accuracy	12.0 (2.9)	12.9 (2.7)	11.2 (3.3)	12.2 (3.1)
s6	Creature Counting Speed	10.4 (3.7)	11.8 (2.6)	11.0 (3.5)	11.7 (3.3)
**Sustained-divided attention**					
s7	Sky Search DT Decrement	8.0 (3.7)	9.3 (3.7)	8.5 (4.2)	8.8 (3.7)

**TABLE 3:** Difference in Time condition between pre- and post-test during the music and video game interventions.

**TEA-Ch subscores**		**Music intervention (*n* = 29)**				**Video game intervention (*n* = 29)**			
		***p*-value of ANOVA**	***p*-value of ANCOVA**			***p*-value of ANOVA**	***p*-value of ANCOVA**		
			**RCPM**	**ADHD-RS**	**RCPM + ADHD-RS**		**RCPM**	**ADHD-RS**	**RCPM + ADHD-RS**
**Selective/focused attention**									
s1	Sky Search Accuracy	0.001**	0.131	0.008**	0.01*	0.001**	0.131	0.007**	0.006**
s2	Sky Search Time per Target	0.001**	0.131	0.008**	0.01*	0.001**	0.131	0.007**	0.006**
s3	Sky Search Attention Score	0.001**	0.131	0.008**	0.01*	0.001**	0.131	0.007**	0.006**
**Sustained attention**									
s4	Score! Accuracy	0.514	0.646	0.956	0.892	0.514	0.646	0.956	0.892
**Attention control/switching**									
s5	Creature Counting Accuracy	< 0.001***	0.128	0.011*	0.011*	< 0.001***	0.797	0.011*	0.011*
s6	Creature Counting Speed	< 0.001***	0.003**	0.011*	0.011*	< 0.001***	0.797	0.011*	0.011*
**Sustained-divided attention**									
s7	Sky Search DT Decrement	0.194	0.086	0.379	0.35	0.194	0.086	0.379	0.35

In this study, we applied ANOVA and ANCOVA using participants’ RCPM score, ADHD-RS score, and RCPM × ADHD-RS scores as covariates. Significant differences between pre- and post-test were found in both the music and video game interventions. Notably, the intervention effects were modulated by participants’ IQ traits; specifically, when controlling for RCPM score, “Creature Counting Speed” was facilitated by the music intervention but not by the video game intervention during attention control/switching. ***p < 0.001; **p < 0.01; *p < 0.05.

### Sky Search Accuracy, Time per Target, and Attention Score

#### ANOVAs

**FIGURE 1:**
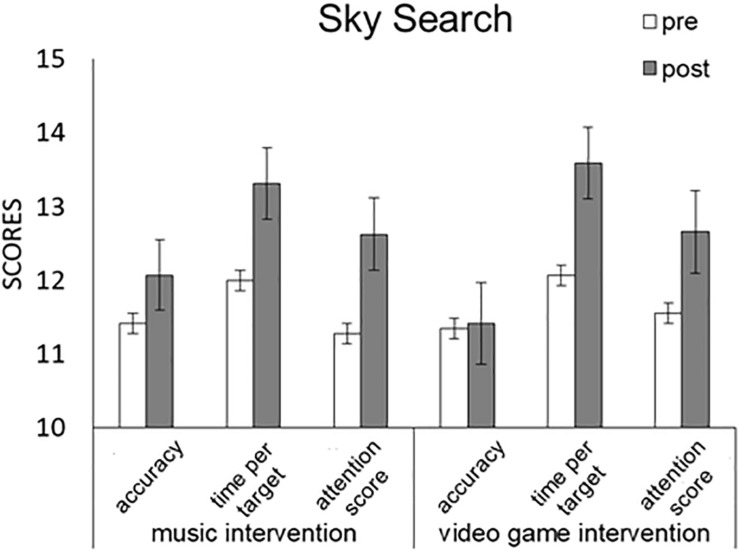
Comparison of mean (with SE) attention score of TEA-Ch between pre and post music and video game interventions in “Sky Search.” Error bars represent standard errors.

We conducted a Task (music, game) × Time (pre, post) × TEA-Ch Score (s1, s2, s3) repeated-measures ANOVA (Figure 1 and Table 3). Significant main effects were detected for TEA-Ch Score [*F*(1,28) = 10.906, *p* < 0.001, partial η^2^ = 0.447] among 11.56, 12.741, and 12.026, and Time [*F*(1,28) = 15.321, *p* = 0.001, partial η^2^ = 0.354] with scores from pre (11.609) and post (12.609), but not for Task [*F*(1,28) = 0.001, *p* = 0.979, partial η^2^ < 0.001] with scores from pre (12.115) and post (12.103). The results thus indicated that selective/focused attention was facilitated by the music and video game interventions.

#### ANCOVAs

We used participants’ RCPM score as a covariate, and conducted a Task (music, game) × Time (pre, post) × TEA-Ch Score (s1, s2, s3) repeated-measures ANCOVA (Table 3). Significant main effect was detected for TEA-Ch Score [*F*(1,27) = 0.23, *p* = 0.796, partial η^2^ = 0.017]. No significant main effects were detected for Task [*F*(1,27) = 0.236, *p* = 0.631, partial η^2^ = 0.009] or Time [*F*(1,27) = 2.422, *p* = 0.131, partial η^2^ = 0.082], and no significant interaction effects were detected for Task × RCPM [*F*(1,27) = 0.247, *p* = 0.624, partial η^2^ = 0.009], Time × RCPM [*F*(1,27) = 0.846, *p* = 0.366, partial η^2^ = 0.03], TEA-Ch × RCPM [*F*(1,27) = 0.388, *p* = 0.682, partial η^2^ = 0.029], Task × Time × RCPM [*F*(1,27) = 0.092, *p* = 0.764, partial η^2^ = 0.003], Task × TEA-Ch × RCPM [*F*(1,27) = 0.158, *p* = 0.855, partial η^2^ = 0.012], Time × TEA-Ch × RCPM [*F*(1,27) = 0.297, *p* = 0.746, partial η^2^ = 0.022], or Task × Time × TEA-Ch × RCPM [*F*(1,27) = 1.327, *p* = 0.283, partial η^2^ = 0.093]. The results thus indicated that, when controlling for IQ traits, selective/focused attention was not modulated by the music or video game interventions.

Moreover, we used participants’ ADHD-RS score as a covariate, and conducted a Task (music, game) × Time (pre, post) × TEA-Ch Score (s1, s2, s3) repeated-measures ANCOVA (Table 3). Although no significant main effect was detected for Task [*F*(1,27) = 2.082, *p* = 0.161, partial η^2^ = 0.072] and no significant interaction effects were detected for Task × ADHD-RS [*F*(1,27) = 3.727, *p* = 0.064, partial η^2^ = 0.121], Time × ADHD-RS [*F*(1,27) = 0.616, *p* = 0.439, partial η^2^ = 0.022], TEA-Ch × ADHD-RS [*F*(1,27) = 0.178, *p* = 0.838834, partial η^2^ = 0.013], Task × TEA-Ch × ADHD-RS [*F*(1,27) = 1.288, *p* = 0.293, partial η^2^ = 0.09], Time × TEA-Ch × ADHD-RS [*F*(1,27) = 0.025, *p* = 0.976, partial η^2^ = 0.002], or Task × Time × TEA-Ch × ADHD-RS [*F*(1,27) = 0.565, *p* = 0.575, partial η^2^ = 0.042], a significant interaction effect was detected for Task × Time × ADHD-RS [*F*(1,27) = 7.229, *p* = 0.012, partial η^2^ = 0.211].

A *post hoc* test showed that, when controlling for the ADHD-RS score, selective/focused attention was facilitated by the music intervention (*p* = 0.008) with scores from pre (11.563) and post (12.667) and by the video game intervention (*p* = 0.007) with scores from pre (11.655) and post (12.552). The results showed that, when controlling for ADHD traits, “accuracy,” “time per target,” and “attention score” were facilitated by the music and video game interventions during selective/focused attention.

Finally, we used participants’ RCPM score × ADHD-RS score as covariates, and conducted a Task (music, game) × Time (pre, post) × TEA-Ch Score (s1, s2, s3) repeated-measures ANCOVA (Table 3). Although no significant main effect was detected for Task [*F*(1,27) = 2.016, *p* = 0.167, partial η^2^ = 0.069] and no significant interaction effects were detected for Task × RCPM × ADHD-RS [*F*(1,27) = 3.689, *p* = 0.065, partial η^2^ = 0.12], Time × RCPM × ADHD-RS [*F*(1,27) = 0.36, *p* = 0.553, partial η^2^ = 0.013], TEA-Ch × RCPM × ADHD-RS [*F*(1,27) = 0.077, *p* = 0.926, partial η^2^ = 0.006], Task × TEA-Ch × RCPM × ADHD-RS [*F*(1,27) = 0.112, *p* = 0.341, partial η^2^ = 0.079], Time × TEA-Ch × RCPM × ADHD-RS [*F*(1,27) = 0.032, *p* = 0.969, partial η^2^ = 0.002], or Task × Time × TEA-Ch × RCPM × ADHD-RS [*F*(1,27) = 1.036, *p* = 0.369, partial η^2^ = 0.074], significant main effects were detected for TEA-Ch Score [*F*(1,27) = 4.968, *p* = 0.015, partial η^2^ = 0.277] and Time [*F*(1,27) = 4.876, *p* = 0.036, partial η^2^ = 0.153] and a significant interaction effect was detected for Task × Time × RCPM × ADHD-RS [*F*(1,27) = 6.371, *p* = 0.018, partial η^2^ = 0.191].

A *post hoc* test showed that, when controlling for IQ and ADHD traits (RCPM and ADHD scores), selective/focused attention was facilitated by the music intervention (*p* = 0.01) with scores from pre (11.567) and post (12.655) and the video game intervention (*p* = 0.006) with scores from pre (11.631) and post (12.536). The results showed that, when simultaneously controlling for IQ and ADHD traits, “accuracy,” “time per target,” and “attention score” were facilitated by the music and video game interventions during selective/focused attention.

### Score! Accuracy

#### ANOVAs

**FIGURE 2:**
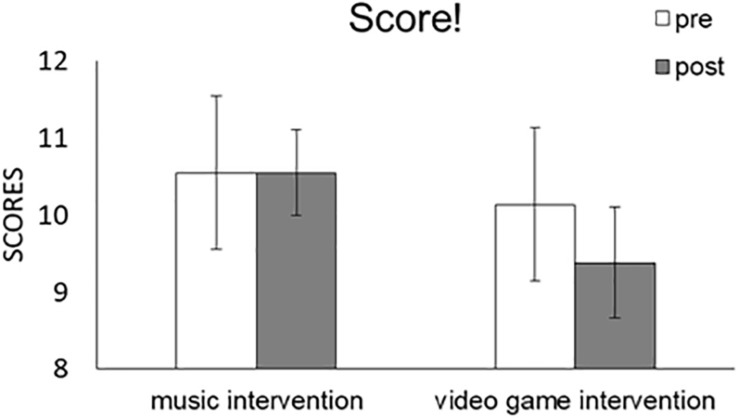
Comparison of mean (with SE) attention score of TEA-Ch between pre and post music and video game interventions in ‘Score!’. Error bars represent standard errors.

We conducted a Task (music, game) × Time (pre, post) × TEA-Ch Score (s4) repeated-measures ANOVA (Figure 2 and Table 3). No significant main effects were detected for Task [*F*(1,28) = 3.119, *p* = 0.088, partial η^2^ = 0.1] with scores from pre (10.552) and post (9.759) or Time [*F*(1,28) = 0.438, *p* = 0.514, partial η^2^ = 0.015] with scores from pre (10.345) and post (9.966). Moreover, no significant interaction effect was detected for Task × Time [*F*(1,28) = 0.921, *p* = 0.345, partial η^2^ = 0.032]. The results thus indicated that sustained attention was not modulated by the music or video game interventions.

#### ANCOVAs

We used participants’ RCPM score as a covariate, and conducted a Task (music, game) × Time (pre, post) × TEA-Ch Score (s4) repeated-measures ANCOVA (Table 3). No significant main effects were detected for Task [*F*(1,27) = 0.163, *p* = 0.689, partial η^2^ = 0.006] or Time [*F*(1,27) = 0.215, *p* = 0.646, partial η^2^ = 0.008], and no significant interaction effects were detected for Task × RCPM [*F*(1,27) = 0.014, *p* = 0.908, partial η^2^ = 0.001], Time × RCPM [*F*(1,27) = 0.337, *p* = 0.566, partial η^2^ = 0.012], or Task × Time × RCPM [*F*(1,27) = 0.96, *p* = 0.336, partial η^2^ = 0.034]. The results thus indicated that, when controlling for IQ traits, sustained attention was not modulated by the music or video game interventions.

Moreover, we used participants’ ADHD-RS score as a covariate, and conducted a Task (music, game) × Time (pre, post) × TEA-Ch Score (s4) repeated-measures ANCOVA (Table 3). No significant main effects were detected for Task [*F*(1,27) = 0.1582, *p* = 0.219, partial η^2^ = 0.055] or Time [*F*(1,27) = 0.003, *p* = 0.956, partial η^2^ < 0.001], and no significant interaction effects were detected for Task × ADHD-RS [*F*(1,27) = 0.014, *p* = 0.907, partial η^2^ = 0.001], Time × ADHD-RS [*F*(1,27) = 0.272, *p* = 0.606, partial η^2^ = 0.01], or Task × Time × ADHD-RS [*F*(1,27) = 0.153, *p* = 0.699, partial η^2^ = 0.006]. The results indicated that, when controlling for ADHD traits, sustained attention was not modulated by the music or video game interventions.

Finally, we used participants’ RCPM score × ADHD-RS score as covariates, and conducted a Task (music, game) × Time (pre, post) × TEA-Ch Score (s4) repeated-measures ANCOVA (Table 3). No significant main effects were detected for Task [*F*(1,27) = 1.861, *p* = 0.184, partial η^2^ = 0.064] or Time [*F*(1,27) = 0.019, *p* = 0.892, partial η^2^ = 0.001], and no significant interaction effects were detected for Task × RCPM × ADHD-RS [*F*(1,27) = 0.059, *p* = 0.811, partial η^2^ = 0.002], Time × RCPM × ADHD-RS [*F*(1,27) = 0.179, *p* = 0.676, partial η^2^ = 0.007], or Task × Time × RCPM × ADHD-RS [*F*(1,27) = 0.151, *p* = 0.701, partial η^2^ = 0.006]. The results indicated that, when simultaneously controlling for IQ and ADHD traits, sustained attention was not modulated by the music or video game interventions.

### Creature Counting Accuracy and Speed

#### ANOVAs

**FIGURE 3:**
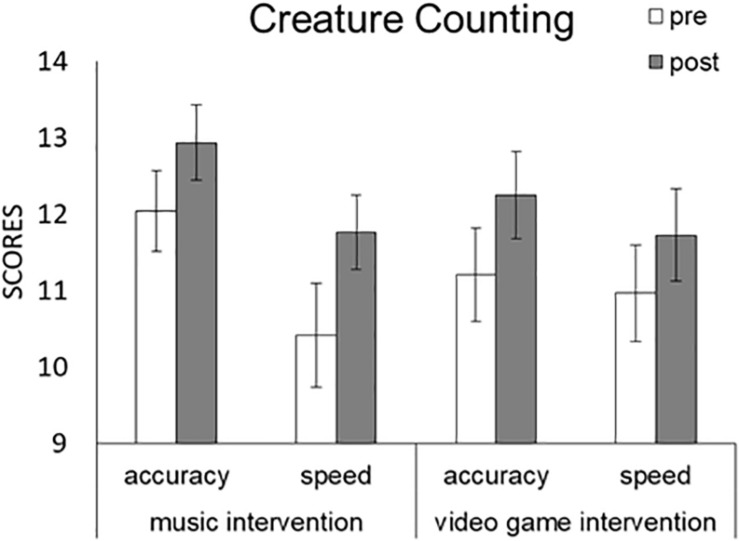
Comparison of mean (with SE) attention score of TEA-Ch between pre and post music and video game interventions in “Creature Counting.” Error bars represent standard errors.

We conducted a Task (music, game) × Time (pre, post) × TEA-Ch Score (s5, s6) repeated-measures ANOVA (Figure 3 and Table 3). A significant main effect was detected for Time [*F*(1,28) = 20.429, *p* < 0.001, partial η^2^ = 0.422] with pre (11.155) and post (12.164) scores, but no significant main effects were detected for Task [*F*(1,28) = 0.308, *p* = 0.583, partial η^2^ = 0.011] with pre (11.784) and post (11.534) scores or TEA-Ch [*F*(1,28) = 2.071, *p* = 0.161, partial η^2^ = 0.069] with pre (12.103) and post (11.216) scores. The results indicated that attention control/switching was facilitated by the music and video game interventions.

However, no significant interaction effects were detected for Task × Time [*F*(1,28) = 0.169, *p* = 0.684, partial η^2^ = 0.006], Task × TEA-Ch [*F*(1,28) = 2.355, *p* = 0.136, partial η^2^ = 0.078], Time × TEA-Ch [*F*(1,28) = 0.029, *p* = 0.867, partial η^2^ = 0.001], or Task × Time × TEA-Ch [*F*(1,28) = 0.789, *p* = 0.382, partial η^2^ = 0.027].

#### ANCOVAs

We used participants’ RCPM score as a covariate, and conducted a Task (music, game) × Time (pre, post) × TEA-Ch Score (s5, s6) repeated-measures ANCOVA (Table 3). Although no significant main effects were detected for Task [*F*(1,27) = 0.248, *p* = 0.623, partial η^2^ = 0.009] or Time [*F*(1,27) = 0.429, *p* = 0.518, partial η^2^ = 0.016], and no significant interaction effects were detected for Task × RCPM [*F*(1,27) = 0.357, *p* = 0.555, partial η^2^ = 0.013], Time × RCPM [*F*(1,27) = 0.007, *p* = 0.933, partial η^2^ < 0.001], Task × Time × RCPM [*F*(1,27) = 0.727, *p* = 0.401, partial η^2^ = 0.026], Task × TEA-Ch × RCPM [*F*(1,27) = 0.486, *p* = 0.492, partial η^2^ = 0.018], or Time × TEA-Ch × RCPM [*F*(1,27) = 0.518, *p* = 0.478, partial η^2^ = 0.019], a significant main effect was detected for TEA-Ch Score [*F*(1,27) = 5.241, *p* = 0.03, partial η^2^ = 0.163] and significant interaction effects were detected for TEA-Ch × RCPM [*F*(1,27) = 4.265, *p* = 0.049, partial η^2^ = 0.136] and Task × Time × TEA-Ch × RCPM [*F*(1,27) = 8.47, *p* = 0.007, partial η^2^ = 0.239].

In the music intervention condition, we used participants’ RCPM score as a covariate, and conducted a Time (pre, post) × TEA-Ch Score (s5, s6) repeated-measures ANCOVA. A significant main effect was detected for TEA-Ch Score [*F*(1,27) = 7.184, *p* = 0.012, partial η^2^ = 0.21] and significant interaction effects were detected for TEA-Ch × RCPM [*F*(1,28) = 5.457, *p* = 0.027, partial η^2^ = 0.168] and Task × TEA-Ch × RCPM [*F*(1,28) = 4.812, *p* = 0.037, partial η^2^ = 0.151]. No significant main effect was detected for Time [*F*(1,27) = 5.457, *p* = 0.23, partial η^2^ = 0.053], and no significant interaction effects were detected for TEA-Ch × RCPM [*F*(1,28) = 5.457, *p* = 0.027, partial η^2^ = 0.168] or Time × RCPM [*F*(1,28) = 0.439, *p* = 0.513, partial η^2^ = 0.016]. A *post hoc* test showed that, when controlling for IQ traits (RCPM score), “speed” (*p* = 0.003), with scores from pre (12.034) and post (12.931) (*p* = 0.128), but not “accuracy” (*p* = 0.081), with scores from pre (10.414) and post (11.759) (*p* = 0.128), was facilitated by the music intervention during attention control/switching. The results indicated that, when controlling for IQ traits, “speed” was facilitated by the music intervention during attention control/switching.

In the video game intervention condition, we used participants’ RCPM score as a covariate, and conducted a Time (pre, post) × TEA-Ch Score (s5, s6) repeated-measures ANCOVA. No significant main effects were detected for Time [*F*(1,27) = 0.068, *p* = 0.797, partial η^2^ = 0.002] or TEA-Ch Score [*F*(1,27) = 1.985, *p* = 0.17, partial η^2^ = 0.068], and no significant interaction effects were detected for Time × RCPM [*F*(1,28) = 0.43, *p* = 0.518, partial η^2^ = 0.016], TEA-Ch × RCPM [*F*(1,28) = 1.798, *p* = 0.191, partial η^2^ = 0.062], or Time × TEA-Ch × RCPM [*F*(1,28) = 1.261, *p* = 0.271, partial η^2^ = 0.045]. The results indicated that “accuracy” and “speed” were not facilitated by the video game intervention during attention control/switching.

We used participants’ ADHD-RS score as a covariate and conducted a Task (music, game) × Time (pre, post) × TEA-Ch Score (s5, s6) repeated-measures ANCOVA (Table 3). A significant main effect was detected for Time [*F*(1,27) = 7.53, *p* = 0.011, partial η^2^ = 0.218). No significant main effects were detected for Task (*F* (1, 27) = 1.693, *p* = 0.204, partial η^2^ = 0.059] or TEA-Ch Score [*F*(1,27) = 1.858, *p* = 0.184, partial η^2^ = 0.064], and no significant interaction effects were detected for Task × ADHD-RS [*F*(1,27) = 1.564, *p* = 0.222, partial η^2^ = 0.055], Time × ADHD-RS [*F*(1,27) = 0.123, *p* = 0.729, partial η^2^ = 0.005], TEA-Ch × ADHD-RS [*F*(1,27) = 0.3, *p* = 0.589, partial η^2^ = 0.011], Task × Time × ADHD-RS [*F*(1,27) = 0.407, *p* = 0.529, partial η^2^ = 0.015], Task × TEA-Ch × ADHD-RS [*F*(1,27) = 0.816, *p* = 0.374, partial η^2^ = 0.029], Time × TEA-Ch × ADHD-RS [*F*(1,27) < 0.001, *p* = 0.991, partial η^2^ < 0.001], or Task × Time × TEA-Ch × ADHD-RS [*F*(1,27) = 1.135, *p* = 0.296, partial η^2^ = 0.04]. The results indicated that, when controlling for ADHD traits, “accuracy” and “speed” were not modulated by the music and video game interventions during attention control/switching.

We used participants’ RCPM score × ADHD-RS score as covariates, and conducted a Task (music, game) × Time (pre, post) × TEA-Ch Score (s5, s6) repeated-measures ANCOVA (Table 3). A significant main effect was detected for Time [*F*(1,27) = 7.364, *p* = 0.011, partial η^2^ = 0.214]. No significant main effects were detected for Task [*F*(1,27) = 1.573, *p* = 0.221, partial η^2^ = 0.055] or TEA-Ch Score [*F*(1,27) = 2.252, *p* = 0.145, partial η^2^ = 0.077], and no significant interaction effects were detected for Task × RCPM × ADHD-RS [*F*(1,27) = 1.428, *p* = 0.242, partial η^2^ = 0.05], Time × RCPM × ADHD-RS [*F*(1,27) = 0.209, *p* = 0.651, partial η^2^ = 0.008], TEA-Ch × RCPM × ADHD-RS [*F*(1,27) = 0.517, *p* = 0.478, partial η^2^ = 0.019], Task × Time × RCPM × ADHD-RS [*F*(1,27) = 0.28, *p* = 0.601, partial η^2^ = 0.01], Task × TEA-Ch × RCPM × ADHD-RS [*F*(1,27) = 0.556, *p* = 0.462, partial η^2^ = 0.02], Time × TEA-Ch × RCPM × ADHD-RS [*F*(1,27) = 0.013, *p* = 0.911, partial η^2^ < 0.001], or Task × Time × TEA-Ch × RCPM × ADHD-RS [*F*(1,27) = 0.279, *p* = 0.601, partial η^2^ = 0.01]. The results indicated that, when simultaneously controlling for IQ and ADHD traits, “accuracy” and “speed” were not modulated by the music and video game interventions during attention control/switching.

### Sky Search DT Decrement

#### ANOVAs

**FIGURE 4:**
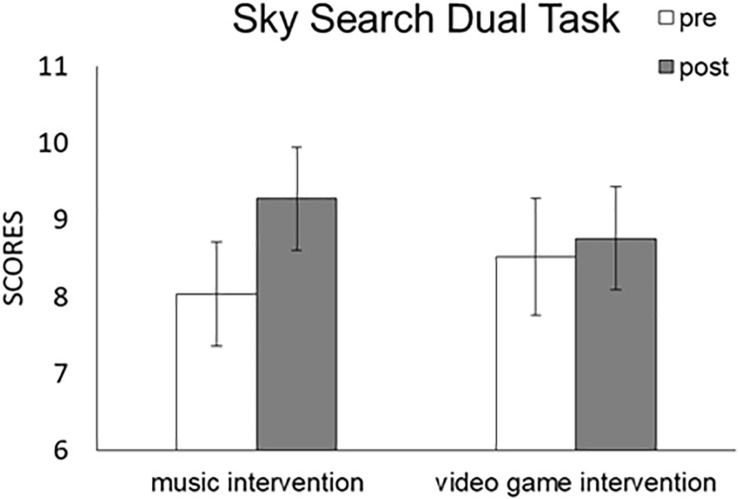
Comparison of mean (with SE) attention score of TEA-Ch between pre and post music and video game interventions in “Sky Search DT.” Error bars represent standard errors.

We conducted a Task (music, game) × Time (pre, post) × TEA-Ch Score (s7) repeated-measures ANOVA (Figure 4 and Table 3). No significant main effects were detected for Task [*F*(1,28) = 0.001, *p* = 0.977, partial η^2^ < 0.001] with pre (8.655) and post (8.638) scores or Time [*F*(1,28) = 1.772, *p* = 0.194, partial η^2^ = 0.06] with pre (8.276) and post (9.017) scores. Moreover, no significant interaction effect was detected for Task × Time [*F*(1,28) = 1.331, *p* = 0.258, partial η^2^ = 0.045]. The results indicated that sustained-divided attention was not modulated by the music or video game interventions.

#### ANCOVAs

We used participants’ RCPM score as a covariate and conducted a Task (music, game) × Time (pre, post) × TEA-Ch Score (s7) repeated-measures ANCOVA (Table 3). No significant main effects were detected for Task [*F*(1,27) = 0.062, *p* = 0.805, partial η^2^ = 0.002] or Time [*F*(1,27) = 3.166, *p* = 0.086, partial η^2^ = 0.105], and no significant interaction effects were detected for Task × RCPM [*F*(1,27) = 0.066, *p* = 0.799, partial η^2^ = 0.002], Time × RCPM [*F*(1,27) = 2.477, *p* = 0.127, partial η^2^ = 0.084], or Task × Time × RCPM [*F*(1,27) = 0.527, *p* = 0.474, partial η^2^ = 0.019]. The results indicated that, when controlling for IQ traits, sustained-divided attention was not modulated by the music or video game interventions.

Moreover, we used participants’ ADHD-RS score as a covariate, and conducted a Task (music, game) × Time (pre, post) × TEA-Ch Score (s7) repeated-measures ANCOVA (Table 3). No significant main effects were detected for Task [*F*(1,27) = 0.093, *p* = 0.763, partial η^2^ = 0.003] or Time [*F*(1,27) = 0.799, *p* = 0.379, partial η^2^ = 0.029], and no significant interaction effects were detected for Task × ADHD-RS [*F*(1,27) = 0.192, *p* = 0.664, partial η^2^ = 0.007], Time × ADHD-RS [*F*(1,27) < 0.001, *p* = 0.988, partial η^2^ < 0.001], or Task × Time × ADHD-RS [*F*(1,27) = 0.005, *p* = 0.943, partial η^2^ < 0.001]. The results indicated that, when controlling for ADHD traits, sustained-divided attention was not modulated by the music or video game interventions.

Finally, we used participants’ RCPM score × ADHD-RS score as covariates, and conducted a Task (music, game) × Time (pre, post) × TEA-Ch Score (s7) repeated-measures ANCOVA (Table 3). No significant main effects were detected for Task [*F*(1,27) = 0.165, *p* = 0.688, partial η^2^ = 0.006] or Time [*F*(1,27) = 0.903, *p* = 0.35, partial η^2^ = 0.032], and no significant interaction effects were detected for Task × RCPM × ADHD-RS [*F*(1,27) = 0.339, *p* = 0.565, partial η^2^ = 0.012], Time × RCPM × ADHD-RS [*F*(1,27) = 0.006, *p* = 0.939, partial η^2^ < 0.001], or Task × Time × RCPM × ADHD-RS [*F*(1,27) = 0.028, *p* = 0.869, partial η^2^ = 0.001]. These results indicated that, when simultaneously controlling for IQ and ADHD traits, sustained-divided attention was not modulated by the music or video game interventions.

## Discussion

The present study examined the effect of a music intervention on healthy children’s attention, using a standardized attention test battery. In the music intervention, we found an enhanced effect on the response speed of attention control/switching when participants’ IQ traits were controlled, compared to the video game intervention. On selective/focused attention, results showed that both music and video game interventions yielded significant improvement, however, the effects disappeared when controlling for participants’ IQ traits. These findings indicate that IQ traits influence attentional performance (Manly et al., 2001; Hurford et al., 2017) and extend our knowledge by demonstrating that IQ traits also influence intervention effects. Notably, there were no effects found for sustained attention and sustained-divided attention under either the music or video game interventions. This is the first study to investigate different types of attention with multiple participants.

Our results show that the music intervention significantly improved children’s attention control/switching compared to the video game intervention, even when controlling for IQ traits. However, when controlling for ADHD traits, the effects disappeared. This may be because ADHD-RS and this subtest of the TEA-Ch reflect common elements of attention. Our finding that the music intervention improved children’s attention control substantiates the previous findings of Pasiali et al. (2014) and Knox et al. (2003) who found positive changes in adolescents with neurodevelopmental delays and brain injury using small sample sizes.

In the present study’s music intervention, participants were asked to play percussion instruments following and/or matching the experimenter’s singing or keyboard playing. During the music intervention, participants found it necessary to switch their attention auditorily and visually between the experimenter’s singing or playing and their own playing while following the tempo, rhythm, and listening to the melody provided by the experimenter (Thaut and Gardiner, 2014; Gfeller, 2008). Attention control is defined as ‘the ability to exert effortful control in order to inhibit a dominant response, to hold in working memory newly relevant rules that require the suppression or activation of previously learned responses, and to shift attention between tasks’ (Cornish and Wilding, 2010, p. 372). When participants played three kinds of percussion instruments to music, they needed to shift their attention to play the instruments while following musical cues from the experimenter. The musical cues were multi-layered and changed randomly; thus, throughout the activity, participants needed to rapidly shift their attention back and forth to the rhythm to play along to the beats, to the pitch to change their instrument, and to the volume to change their loudness. That is, it was not adequate to just pay attention to what they were playing on their own instruments; they also needed to change how they played, depending on the pitch, volume, and tempo of the music that was produced by the experimenter and to determine when to start/stop–all of which changed over time.

Active music listening to play along could have required attention control, as participants frequently switched back and forth from each musical element (Lipscomb, 1996). It also required the participants to hold these instructions in working memory; specifically, how to play along while following the musical cues and when to pause playing when the music stopped, which were possibly new rules for them. In sum, referring to the concept of attention control, active participation in the music intervention, while interacting with the experimenter, required participants to: (1) exert effortful control to inhibit steady continuous performance and follow and respond to musical cues that were changing over time; (2) hold in working memory new instructions on how to respond that were possibly different from their previous music experience, which allowed them to simply play along; and (3) shift their attention rapidly between the experimenter/keyboard and their instruments (Thaut and Gardiner, 2014; Lipscomb, 1996; Gfeller, 2008). The music used in this study was structured with multiple elements that included both verticality (simultaneity) and horizontality (sequentiality) at the same time (Thaut, 2005) which may have enhanced participants’ attention control. In conjunction with these possible factors, the unpredictability of the music intervention may be another factor to consider in our results. That is, in the music intervention, participants had to adapt to music stimuli that were provided by the experimenter to respond appropriately over time in the temporal structure. Thus, compared to the video game intervention, in which participants responded to relatively constant requirements and took turns doing so, the music intervention was more unpredictable. The constantly changing nature of the music, including the varied elements, could have impacted attention control. Thus, these components of music may positively influence participants’ attention control; hence, music appears to be a suitable tool for attention control training.

When controlling for IQ traits, the significant intervention effects on the selective attention subtest scores disappeared. The general view is that children with higher IQs score better, and in this case, the children with higher IQs found the targets faster and more accurately. In fact, different searching strategies were observed in participants during test taking. For example, some children darted impulsively from one part of the test sheet to another and others looked systematically through the sheet columns one at a time to find the targets. This may indicate that these differing strategies are influenced by IQ and may affect this subtest score. In other words, this attention subtype may involve IQ factors that the RCPM measures, with some factors influencing attentional performance, depending on the attention subtypes.

With the subtests that measured sustained and divided attention, no significant improvements were found across the music and video game interventions. Regarding sustained attention, our finding is contrary to those of previous studies (Robb, 2003b; Lee, 2006; Wolfe and Noguchi, 2009; Kasuya, 2011). Two possible factors should be considered. First, some studies (Robb, 2003b; Lee, 2006; Wolfe and Noguchi, 2009) investigated participants’ sustained attention during one or more music interventions or conditions, meaning that it is unclear whether the effects can be generalized to other tasks, like attention tests, although children exert higher sustained attention in musical environments. Another possibility is the frequency and period of intervention administration. More specifically Kasuya (2011) observed improvements on a computer-based attention test after 24 sessions across 11 months. In contrast, our finding is based on a single, short intervention. Pasiali et al. (2014) also did not observe changes in sustained attention after eight interventions across 6 weeks. This suggests that longer and more frequent interventions may be necessary to improve children’s sustained attention. It is likely that the interventions we employed did not activate the divided attention subtype.

Data obtained from the various attentional tasks suggest the possibility of cross-modal effects of music intervention on attention. That is, the attention control/switching subtest (“Creature Counting”), which required visual attention, improved participants’ performance after the music intervention. The performance on selective attention subtest (“Sky Search”) improved significantly after both interventions and also required visual attention. However, no significant improvement was found in the auditory sustained attention subtest (‘Score!’) nor in the auditory and visual sustained-divided attention subtest (“Sky Search DT”), following the music intervention, which is inconsistent with previous findings (Degé et al., 2011; Strait and Kraus, 2011). Music interventions likely require more auditory attention, although both interventions required visual attention as well; therefore, our findings did not support our hypothesis that a music intervention would improve auditory attention more than a video game intervention. Nevertheless, our results suggest that attention is not a modality-specific function. Furthermore, because of the multimodality of instrumental music activity (Pantev et al., 2009) even with the low complexity of playing instruments in our study, music interventions with instruments have yielded cross-modal effects (Janzen and Thaut, 2018). However, some previous studies have reported no significant effects of music instrument lessons on visual attention (Strait et al., 2010; Roden et al., 2014) thus, further studies are needed to examine potential cross-modal effects of a simple music activity, especially since previous studies that reviewed the influence of long-term musical training reported that benefits may be restricted to the auditory domain (Roden et al., 2014; Martens et al., 2015).

The emotional and motivational factors associated with music are often addressed when considering the influence of music on children’s cognitive function (Thaut and Gardiner, 2014; Gfeller, 2008). Research findings indicate that these factors help children focus and facilitate learning (Abikoff et al., 1996; Geist and Geist, 2008, 2012; Xu et al., 2010). However, our results suggest that this outcome may be related to both motivational and engaging factors of music and the music components, since the control intervention employed in this study was also motivational for children and contained engaging factors. This result suggests that the music itself, with its components described above, contains factors that enhance attention control in children, since the video game intervention was also fun and child friendly, with an adequate attentional requirement. Observations revealed that the competing scores obtained on each bowling turn with the experimenter were motivational for participants and elicited positive emotions. During the 30-minute intervention, participants seemed to concentrate on the video game intervention as well as they did in the music intervention. Previous studies that have found effects of music on attention in typical children (Morton et al., 1990; Wolfe and Noguchi, 2009) compared music conditions to speech or quiet conditions, which are control conditions that may be less motivational and engaging for children than the video game used here. Thus, the results of the present study may reflect the intrinsic features of the music intervention more than the impact of emotional and motivational factors on attention control.

Previous studies show that longitudinal music training influences at least some non-musical functioning in children (Yang et al., 2014; Linnavalli et al., 2018; Nan et al., 2018; Barbaroux et al., 2019; Fasano et al., 2019; Schellenberg, 2004) however, findings on the enhancing effects on cognitive functions in children have been inconsistent. One factor that has contributed to this inconsistency may be differences in musical content (Dumont et al., 2017) which varies greatly between the studies, from general music programs for young children (Linnavalli et al., 2018) to intense instrumental music lessons (Nan et al., 2018). If the participants’ musical content differs, both musical and non-musical skills and the brain regions and networks used during music engagement likely differ, leading to different results in measured cognitive functions. The present study examined the effects of a short-term music intervention designed for attention training and set a similar non-music intervention as an active control task, with results that contradicted those of some previous studies on music training (Scott, 1992; Rickard et al., 2010; Degé et al., 2011; Roden et al., 2014). For instance, our music intervention resulted in significant improvement in attention control compared to the non-musical intervention. It is possible that differences in musical content may underlie the inconsistencies between the present study results and those of previous studies that focused on music training to learn music and/or instrumental performance skills.

Computerized programs developed specifically for attention training (Tucha et al., 2011; Rueda et al., 2012; Kirk et al., 2016; Spaniol et al., 2018) and Attention Process Training programs adapted for children (Kerns et al., 1999; Tamm et al., 2013) have been extensively studied. In computerized programs, children play games to train various attention skills. However, while studies on computerized programs have shown promising effects on children’s attention, they have yielded mixed findings by attention subtypes, targeted populations, and participants’ ages (Tucha et al., 2011; Rueda et al., 2012; Kirk et al., 2016). Our study shows that interactive music intervention improves attention control/switching when controlling for participants’ IQ traits compared to the active control intervention, but no previous studies on computerized programs have reported similar effects. The intrinsic features of music interventions, such as their highly social aspects (Davis et al., 2008; Wheeler, 2015) may have influenced our results. Behaviors resulting from poor attention skills can cause problems, mostly in social situations such as in the classroom or in educational conversations (DuPaul et al., 1998) thus, interactive music interventions may be beneficial for some children with attentional difficulties. Some researchers have recommended that attention rehabilitation should involve an intervenient to monitor progress, give feedback, and teach strategies (Gardiner, 2005) arguing that stand-alone use of computers may not be appropriate for some children who need attention rehabilitation (Cicerone et al., 2000; Weber, 1990). Given that previous findings are inconsistent regarding attention subtypes, targeted populations and ages, and the intervention’s frequency and duration, future research should investigate which programs benefit specific attention subtypes and children’s functional attention skills to develop evidence-based practices.

This study has a few limitations. First, we targeted 6-to-9-year-old children, whose neuroplasticity would have been relatively high in relation to the overall age range of the TEA-Ch (i.e., 6 to 16 years). Thus, the children’s age may have influenced the results. Second, we caution the reader against generalizing our results, because this study investigated immediate and short-term effects of an individual music intervention on attention skills using a neuropsychological test. It is possible that our results were affected by changes in arousal state, since we administered the retest immediately after the intervention. Since our results represent a first step toward uncovering the short-term effects of a music intervention on typically developing children, further longitudinal studies of attention-targeted music interventions are needed to verify that music positively impacts non-musical attentive behaviors. Future studies should follow the child’s progress on attentional performance and examine whether intervention effects are generalizable to attentional performance in the real world for both typically developing children and children with attentional deficits.

It is also necessary to examine the transfer effects of music interventions beyond the scope of this study to discover whether attention-targeted music interventions influence other functions beyond attention, compared to non-music interventions. Since attention skills underlie higher cognitive functions and could possibly affect overall child development, future studies should examine whether attention control improvements driven by music intervention, as found in the present study, have a long-term influence on other cognitive functions, such as general intelligence and executive function. For those with attentional deficits, especially individuals with ADHD and ASD, who are often observed to have attention control difficulties (Fan, 2013; Townsend et al., 1996; Allen and Courchesne, 2001; Goldstein et al., 2001; Rinehart et al., 2001; Landry and Bryson, 2004; Zwaigenbaum et al., 2005; Cornish and Wilding, 2010) we need to investigate whether the effects of music interventions influence their core symptoms. Given that children with ASD have shown improvements in social communication following interventions to improve attention (Matson et al., 2010; Kerns et al., 2017) transfer effects of music intervention to other domains (i.e., social and communication domains) should also be investigated. Children with ADHD, who potentially have core deficits with tracking the beat of music (Puyjarinet et al., 2017) may benefit from music interventions with rhythm-based activities, such as employed in the present study, over background music intervention (Maloy and Perterson, 2014) because children who can synchronize to rhythm display better attentive behaviors (Khalil et al., 2013). An intervention with simple and easy-to-follow musical activities, like those used in this study, should be feasible to implement for children with cognitive impairments and/or developmental disorders (Pasiali et al., 2014; Abrahams and van Dooren, 2018). These possibilities will be the focus of future studies in music interventions.

Although the study by Rueda et al. (2005) showed that attention network development is subject to interventions during childhood, results from widely implemented attention training programs (e.g., computerized training) have been inconsistent. Our results, coupled with a limited number of previous studies with small sample sizes, show that music interventions may have positive impacts on children’s and adolescents’ attention. Currently, there is no gold standard for attention training and the best and most effective approaches may differ, depending on the child’s age, diagnosis, and severity of the attention deficit. Additionally, in future studies of attention, we recommend that researchers uncover details of participants’ past experiences with the research tasks, since familiarity may influence the outcomes. It is possible that the level of attention and cognitive load may be different for a first-time activity than for an experienced activity.

Finally, another limitation should be noted: implementing music interventions with children requires many competencies, such as musical and teaching skills, and the ability to interact, be motivating, and build good relationships. Given that we only implemented a one-time music intervention, it is likely that there were no effects from the intervener-child relationship, however, the intervener’s competence may also have influenced our study results (Standley, 2000). Thus, in future studies, it will be necessary to investigate whether intervener factors (e.g., years of experience and education history) influence intervention effects.

## Conclusion

Our findings indicate that a music intervention has short-term effects on children’s attention control. This is the first evidence showing that an interactive intervention with live music in which a child plays accessible instruments may benefit attention control. Our results suggest that music intervention may be a promising tool to train attention control in children by eliciting underlying induced oscillatory activity associated with attentional ability and neuroplasticity (Trainor et al., 2009; Thaut and Gardiner, 2014). Given that previous studies have reported similar neurological observations following long-term musical engagement (Schlaug et al., 2005, 2009; Hyde et al., 2009) neuroimaging studies are needed to examine the effects of music on brain activity and to deepen our understanding of how music improves children’s cognitive function. Our findings not only provide evidence for the effectiveness of music intervention, they also provide clues toward understanding its neural mechanisms.

## Data Availability Statement

All datasets generated for this study are included in the article/Supplementary Material.

## Ethics Statement

The studies involving human participants were reviewed and approved by the ethics committee of Kyoto University Graduate School of Medicine in accordance with the Declaration of Helsinki (approval number: E1856), and all methods were implemented in accordance with relevant guidelines and regulations. Written informed consent to participate in this study was provided by the participants’ legal guardian/next of kin.

## Author Contributions

YK-U and MT developed the study methods and design. YK-U collected the data, conducted the experiment, and wrote the manuscript. MT interviewed the participants and their parents. SZ performed the statistical analyses. All authors contributed to the manuscript preparation.

## Conflict of Interest

The authors declare that the research was conducted in the absence of any commercial or financial relationships that could be construed as a potential conflict of interest.
