# Supplementary material for: The Effect of Music Intervention on Attention in Children: Experimental Evidence
Source: Front Neurosci. 2020 Jul 24;14:757. doi: 10.3389/fnins.2020.00757 (PMC7393235; doi:10.3389/fnins.2020.00757)
Supplement: Supplementary file 3 [file Data_Sheet_1.docx]

Supplementary Material

# Supplemental Analysis

We examined whether the difference in attentional performance was influenced by task order during selective/focused attention, sustained attention, attention control/switching, and sustained-divided attention. Score differences were analyzed using a repeated-measures ANCOVA with Task (music, game), Time (pre, post), and TEA-Ch Score (e.g., s1) as within-participant factors and task order as a covariate.

# Supplemental Results

## Sky Search Accuracy, Time per Target, and Attention Score

We used participants’ task order as a covariate, and conducted a Task (music, game) × Time (pre, post) × TEA-Ch Score (s1, s2, s3) repeated-measures ANCOVA. Although no significant interaction effects were detected for Time × Task order (*F* (1, 27) = 3.001, *p* = 0.095, partial η^2^ = 0.1), TEA-Ch × Task order (*F* (1, 27) = 1.245, *p* = 0.305, partial η^2^ = 0.087), Task × Time × Task order (*F* (1, 27) = 3.31, *p* = 0.08, partial η^2^ = 0.109), Time × TEA-Ch × Task order (*F* (1, 27) = 0.114, *p* = 0.893, partial η^2^ = 0.009), or Task × Time × TEA-Ch × Task order (*F* (1, 27) = 0.937, *p* = 0.342, partial η^2^ = 0.034), significant effects were detected for Task × Task order (*F* (1, 27) = 83.15, *p* < 0.001, partial η^2^ = 0.755) and Task × TEA-Ch × Task order (*F* (1, 27) = 25.114, *p* < 0.001, partial η^2^ = 0.659). The results thus indicated that, when controlling for Task order, although the difference in Task and Task × TEA-Ch between music and game scores was modulated by task order during selective/focused attention, the effects of ‘accuracy’, ‘time per target’, and ‘attention score’ were not modulated by the music or video game interventions during selective/focused attention.

## Score! Accuracy

We used participants’ task order as a covariate, and conducted a Task (music, game) × Time (pre, post) × TEA-Ch Score (s4) repeated-measures ANCOVA. No significant interaction effects were detected for Task × Task order (*F* (1, 27) = 0.543, *p* = 0.468, partial η^2^ = 0.02), Time × Task order (*F* (1, 27) = 0.596, *p* = 0.447, partial η^2^ = 0.022), or Task × Time × Task order (*F* (1, 27) = 0.383, *p* = 0.541, partial η^2^ = 0.014). The results indicated that, when controlling for Task order, sustained attention was not modulated by the music or video game interventions.

## Creature Counting Accuracy and Speed

We used participants’ task order as a covariate, and conducted a Task (music, game) × Time (pre, post) × TEA-Ch Score (s5, s6) repeated-measures ANCOVA. Although no significant interaction effects were detected for Time × Task order (*F* (1, 27) = 0.785, *p* = 0.384, partial η^2^ = 0.028), TEA-Ch × Task order (*F* (1, 27) = 0.036, *p* = 0.852, partial η^2^ = 0.001), Time × TEA-Ch × Task order (*F* (1, 27) = 0.803, *p* = 0.378, partial η^2^ = 0.029) or Task × Time × TEA-Ch × Task order (*F* (1, 27) = 0.018, *p* = 0.896, partial η^2^ = 0.001), significant effects were detected for Task × Task order (*F* (1, 27) = 36.032, *p* < 0.001, partial η^2^ = 0.575), Task × TEA-Ch × Task order (*F* (1, 27) = 7.614, *p* = 0.01, partial η^2^ = 0.22), and Task × Time × Task order (*F* (1, 27) = 20.723, *p* < 0.001, partial η^2^ = 0.434). A post-hoc test showed that, when controlling for Task order, attention control/switching was facilitated by the music intervention with the scores from pre (11.224) and post (12.345) (*p* < 0.001) and the video game intervention with the scores from pre (11.086) and post (11.983) (*p* = 0.019). The results indicated that, when controlling for Task order, the effects of ‘accuracy’ and ‘speed’ were facilitated by the music and video game interventions during attention control/switching. Given that facilitated effects of ‘accuracy’ and ‘speed’ were also found for both interventions using ANOVA, we suggest that the effects of ‘accuracy’ and ‘speed’ by the music and video game interventions were not modulated even when controlling for Task order during attention control/switching.

## Sky Search Dual Task Decrement

We used participants’ task order as a covariate, and conducted a Task (music, game) × Time (pre, post) × TEA-Ch Score (s7) repeated-measures ANCOVA. Although no significant interaction effects were detected for Time × Task order (*F* (1, 27) = 0.723, *p* = 0.403, partial η^2^ = 0.026) or Task × Time × Task order (*F* (1, 27) = 0.224, *p* = 0.64, partial η^2^ = 0.008), a significant effect was detected for Task × Task order (*F* (1, 27) = 4.438, *p* = 0.045, partial η^2^ = 0.141). The results indicated that, when controlling for Task order, although the Task difference between music and game scores was modulated by Task order during sustained-divided attention, the effect of sustained-divided attention was not modulated by the music or the video game intervention.
